# Disease severity in patients with visceral leishmaniasis is not altered by co-infection with intestinal parasites

**DOI:** 10.1371/journal.pntd.0005727

**Published:** 2017-07-21

**Authors:** Fitsumbrhan Tajebe, Mulusew Getahun, Emebet Adem, Asrat Hailu, Mulualem Lemma, Helina Fikre, John Raynes, Aschalew Tamiru, Zemenay Mulugeta, Ermias Diro, Frederic Toulza, Ziv Shkedy, Tadesse Ayele, Manuel Modolell, Markus Munder, Ingrid Müller, Yegnasew Takele, Pascale Kropf

**Affiliations:** 1 Department of Immunology and Molecular Biology, University of Gondar, Gondar, Ethiopia; 2 Leishmaniasis Research and Treatment Centre, University of Gondar, Gondar, Ethiopia; 3 Department of Microbiology, Immunology and Parasitology, Addis Ababa University, Addis Ababa, Ethiopia; 4 Department of Internal Medicine, University of Gondar, Gondar, Ethiopia; 5 Department of Immunology and Infection, London School of Hygiene and Tropical Medicine, London, United Kingdom; 6 Department of Haematological Medicine, Faculty of Life Sciences, King’s College, London, United Kingdom; 7 Department of Mathematics and Statistics, University of Hasselt, Hasselt, Belgium; 8 Department of Cellular Immunology, Max-Planck-Institute for Immunobiology and Epigenetics, Freiburg, Germany; 9 Third Department of Medicine (Hematology, Oncology, and Pneumology), University Medical Center Mainz, Mainz, Germany; 10 Department of Medicine, Imperial College London, London, United Kingdom; National Institutes of Health, UNITED STATES

## Abstract

Visceral leishmaniasis (VL) is a neglected tropical disease that affects the poorest communities and can cause substantial morbidity and mortality. Visceral leishmaniasis is characterized by the presence of *Leishmania* parasites in the spleen, liver and bone marrow, hepatosplenomegaly, pancytopenia, prolonged fever, systemic inflammation and low body mass index (BMI). The factors impacting on the severity of VL are poorly characterized. Here we performed a cross-sectional study to assess whether co-infection of VL patients with intestinal parasites influences disease severity, assessed with clinical and haematological data, inflammation, cytokine profiles and BMI. Data from VL patients was similar to VL patients co-infected with intestinal parasites, suggesting that co-infection of VL patients with intestinal parasites does not alter disease severity.

## Author summary

Visceral leishmaniasis (VL), a disease caused by a parasite, *Leishmania*, belongs to the most neglected tropical diseases: they mainly occur in low-income countries and affect the poorest populations. The parasites are transmitted via the bite of an insect vector and migrate to the internal organs. When VL occurs, the patients will present with enlarged spleen and liver, a disturbed haematological profile with low blood cell counts, systemic inflammation and malnutrition. This stage of the disease is fatal if left untreated. The factors that influence VL disease severity are poorly characterized. Here, we recruited patients with VL in an area of Ethiopia with a high prevalence of intestinal parasites. Our aim was to assess whether the disease was more severe in VL patients co-infected with intestinal parasites. Our results show that clinical and haematological data, inflammation, cytokine profile and nutritional status of VL patients are similar in VL patients and in VL patients co-infected with intestinal parasites. These results suggest that co-infection of VL patients with intestinal parasites does not impact on disease severity.

## Introduction

The leishmaniases are a group of neglected tropical diseases (NTDs), largely affecting the least developed regions of the world. There is an estimated 350 million people in 98 countries at risk of leishmaniasis [1, 2]. Leishmaniases are a spectrum of diseases: presentations vary from subclinical infection through a range of dermal presentations of varying severity (cutaneous leishmaniasis, CL) to visceral leishmaniasis (VL). VL is caused by *Leishmania* (*L*.) *donovani* or *L*. *infantum* and is the most severe form of the disease. An estimated 200,000 to 400,000 new cases of VL with an incidence of 50,000 deaths occur each year, however these numbers are widely acknowledged to be a gross underestimation of the real burden [3, 4]. In global estimates, Sudan, South Sudan, Ethiopia, Kenya and Somalia account for the second largest number of annual VL cases, after South Asia [3]. VL inflicts an immense toll on the developing world and impedes economic development, with an estimated loss of 2.3 million disability-adjusted life years. There is no effective vaccine; currently used chemotherapy is toxic and increasing drug resistance is reported [5]. Infection with *Leishmania* parasites can be asymptomatic or can manifest as a progressive disease. VL is characterised by hepatosplenomegaly, fever, weight loss, hyperglobulinemia and pancytopenia [6]; if left untreated, it is almost always fatal. In Ethiopia, VL is caused by *L*. *donovani* and it is one of the most significant vector-borne diseases; Ethiopia has the second largest number of VL cases in sub-Saharan Africa with an estimated annual burden of 4500 to 5000 new cases. VL is worsened by malnutrition and HIV co-infection, and it has been suggested that intestinal parasitic infections might also impact on disease severity by modulating cell-mediated immunity and by worsening malnutrition [6]. Helminth infections are characterised by a strong Th2 response [7] and it has been suggested that this might suppress a protective Th1 response in VL patients and therefore contribute to the strong immunosuppression characteristic of these patients [8]. In addition, intestinal parasites may also contribute to malnutrition by competing for nutrients in the gut, inducing chronic inflammation and causing malabsorption. The Northwest of Ethiopia, where the current study took place, has a high prevalence of intestinal parasitic infections (both protozoa and helminths) [6] and malnutrition appears to be relatively common [9]. However, precise information about the impact of co-infection with intestinal parasites on the severity of patients with VL is scarce.

In the current study, we measured the impact of intestinal parasite co-infections on the disease status of patients with VL, before the start of anti-leishmanial treatment. Clinical data were collected and haematological data, inflammatory mediators and cytokines were determined. All these parameters were compared between patients presenting with VL and VL patients co-infected with intestinal parasites.

## Methods

### Subjects and sample collection

The study was approved by the Institutional Review Board of the University of Gondar (IRB, reference SBMLS/1199/07). For this cross-sectional study, a cohort of 42 male non-endemic healthy controls were recruited amongst the staff of Gondar University Hospital and 60 male VL patients were recruited from the Leishmaniasis Research and Treatment Center of Gondar University Hospital before treatment. All patients were male and migrant workers, and indeed the large majority of VL patients at the Leishmaniasis Research and Treatment Center are migrant workers. No women presented with visceral leishmaniasis during our study.

The exclusion criteria were age (<18 years), and co-infection with tuberculosis, malaria and HIV. The diagnosis of VL was based on positive serology (rK39) and the presence of *Leishmania* amastigotes in spleen or bone marrow aspirates [10]. Written Informed consent was obtained from each patient and control. Patients were treated with a combination of sodium stibogluconate (20mg/kg body weight/day), and paromomycin (15mg/kg body weight/day) injections, given intramuscularly for 17 days or with Ambisome^®^ (max of 30mg/kg body weight, with 6 injections of 5mg/kg body weight /day) and showed an initial clinical cure rate of 100% after treatment, defined as follows: at the end of successful treatment, patients look improved, afebrile, and usually have a smaller spleen size than on admission and an improved hematological profile.

The diagnosis of intestinal parasites was made in fresh stools, before the start of anti-leishmanial treatment, collected in a clean screw top container. Part of the collected stool was processed using the direct saline wet mount procedure [11, 12]; part was examined using the Kato-katz technique [11, 12] and the rest was processed using the "formol ether concentration" technique [12]. All preparations were examined for the presence of parasites by microscopy, within 30 minutes after collection; each stool sample was examined by two experienced laboratory technicians. Of note, only active intestinal infections were taken into account: *Giardia* and *Entamoeba* cysts were observed in 3 and 5 patients, respectively, however, no trophozoites were detected in their stools and therefore were not categorized as causing an active infection. Six ml of blood was collected in EDTA tubes before the start of treatment and was processed within 10 minutes after collection: following density gradient centrifugation on Histopaque-1077 (Sigma), the plasma was isolated from the top layer and frozen immediately. To count the percentages of eosinophils, a drop of whole blood was smeared onto a glass slide, stained with Giemsa and the percentages of eosinophils per 100 white blood cells were counted microscopically.

The BMI was measured as follows: Weight (kg)/ (height (m)) ^2^. Normal BMI was defined as ≥18.5, moderate malnutrition as BMI = 18.4–16.5 and severe malnutrition as BMI < 16.5.

### Haematological analysis

The haematological profiles were determined by using an automated CELL-DYN1800 Haematology Analyser, USA.

### ELISA/Luminex

The ELISA for the determination of the acute phase C-reactive protein (CRP) was performed as described in [13] (detection limit: 0.3μg/ml). ELISA kits were used for the determination of IFN-γ, IL-6, IL-8 and IL-10 levels (Ready-SET-Go! ELISA Sets) in plasma, according to the manufacturers’ protocol. The detection limits for these ELISA were 4pg/ml, 2pg/ml, 2pg/ml and 32pg/ml, respectively. Elastase and Myeloperoxidase (BioVendor) were used according to the manufacturers’ protocol. The detection limits for these ELISA were 0.2pg/ml and 0.4ng/ml, respectively.

Cytokine analysis for interleukin (IL)-2, -4, -5, -12 and IL -13 was performed by using the Luminex 200 system (USA, Multiplex Map Kit) and the plate was analyzed using the Luminex 100 system. The detection limits for these tests were 2.5pg/ml, 0.1pg/ml, 3.1 pg/ml, 2.5pg/ml and 3pg/ml, respectively.

### Arginase activity

The enzymatic activity of arginase in the plasma was measured as previously described [14]. Briefly, urea concentrations were first determined without the activation and hydrolysis steps; these values were subtracted from those obtained by measuring the urea levels. One unit of enzyme activity is defined as the amount of enzyme that catalyzes the formation of 1 μmol of urea per min.

### Statistical analysis

Data were evaluated for statistical differences using two-tailed Mann-Whitney (GraphPad Prism 6) and differences were considered statistically significant at *p*<0.05. Results are expressed as median± SEM.

## Results

### Clinical data

**Fig 1:**
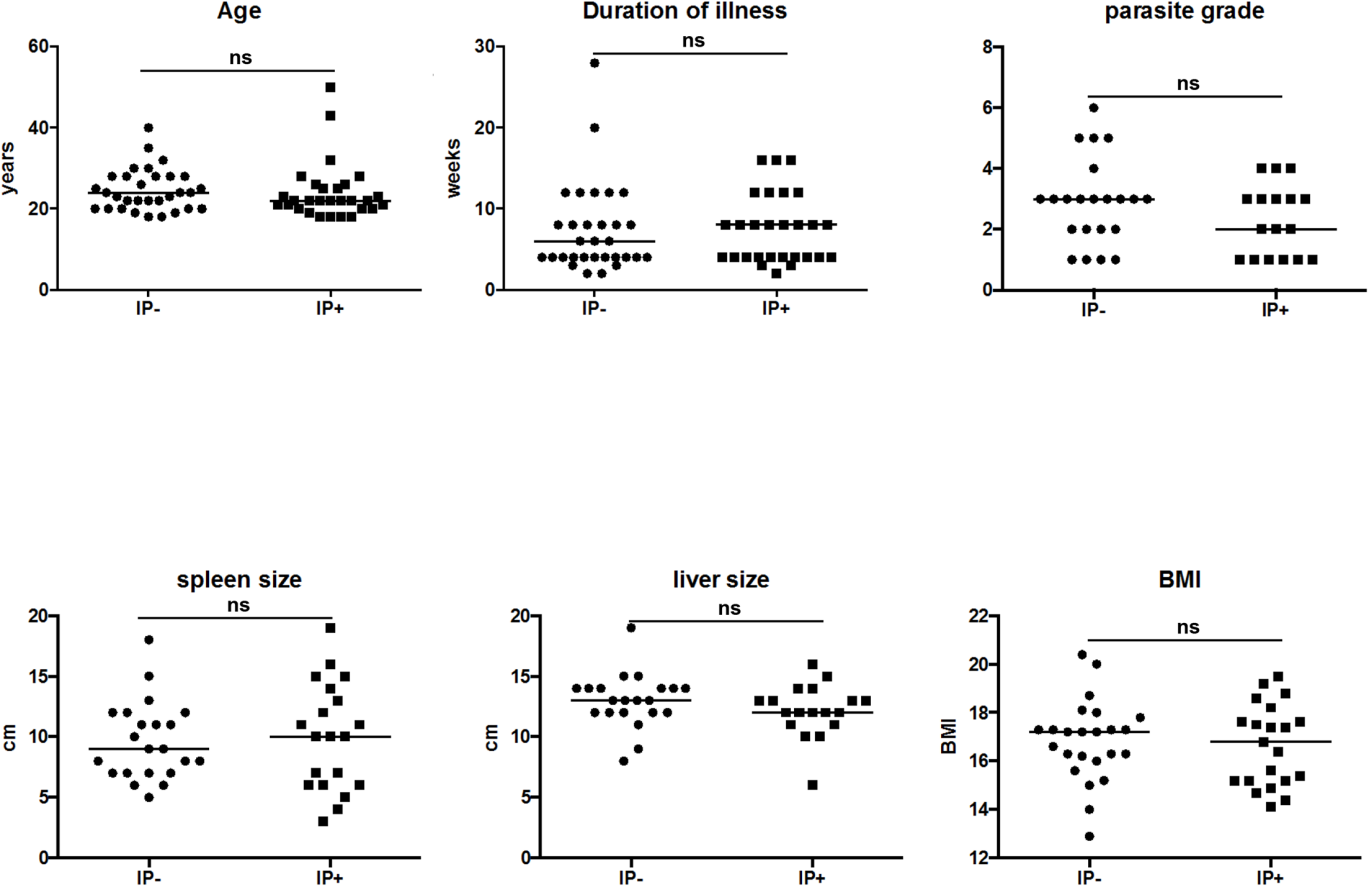
The age, duration of illness (defined as the number of weeks since the patients noticed symptoms associated with visceral leishmaniasis, such as fever, and/or enlarged abdomen (as a sign of enlarged spleen or liver), parasite grades, spleen and liver sizes and BMI was measured as described in Materials and Method in VL patients without intestinal parasite co-infections (IP-) and in VL patients co-infected with intestinal parasites (IP+). Statistical significance was established using a Mann-Whitney test, ns = not significant (*p*<0.05).

Sixty male VL patients were recruited into our study and their clinical data are summarized in Table 1. Their intestinal parasite (IP) status was determined and as shown in Table 2: 48.3% (29 patients = IP+) were positive for intestinal parasites: 18 VL patients were co-infected with hookworms, 8 with *Ascaris* (*A*.) *lumbricoides*, 4 with *Schistosoma*(*S*.) *mansoni*, 2 with *Entamoeba* (*E*.) *histolytica*, 1 with *Trichuris*(*T*.) *trichiura*, and 1 with *Strongyloides* (*S*.) *stercoralis*. Of note, both *S*. *mansoni* and *S*. *haematobium* are prevalent in Ethiopia, mainly in school-age children [15, 16], but since our aim was to study the impact of intestinal parasites on VL disease severity, we focus on *S*. *mansoni*. Out of the 29 VL patients co-infected with intestinal parasites, 5 patients were co-infected with 2 different intestinal parasites (Table 2). When stratified according to their intestinal parasite (IP) status, the two groups were of similar age (IP+: 22.0±1.3 and IP-: 24.0±0.9, p>0.05); had a similar duration of illness (IP+: 8.0±0.8 and IP-: 6.0±1.0 weeks, p>0.05); similar parasite grade (spleen IP+: 3.0±0.2 and IP-: 3.0±0.3, p>0.05); similar spleen size (IP+: 10.0±0.8 and IP-: 10.0±0.7, p>0.05) and liver size (IP+: 12.0±0.5 and IP-: 3.5±0.4, p>0.05); and similar BMI (IP+: 17.4±0.3 and IP-: 17.2±0.3, p>0.05) (Table 1 and Fig 1).

**Table 1:** Clinical data.

Patient	Age	Duration of illness (weeks)	Parasite grade (spleen)	Parasite grade (bone marrow)	Spleen size (cm)	Liver size (cm)	BMI
1	22	12	1		6	11	15.2
2	25	8	5		10	13	16
3	22	4	3		15	15	15.2
4	35	4	1		12	12	20.4
5	43	4	1		15	12	18.2
6	50	8	4		10	13	14.7
7	26	12	1		6	12	16.8
8	22	8	2		7	13	17.3
9	18	12		2	11	16	15.2
10	30	6	2		9	12	17.8
11	21	16	4		11	15	15.2
12	23	8	1		not palpable	14	17.6
13	20	4		1	7	12	14.4
14	28	12	3		8	12	16.3
15	28	4	3		13	14	16.2
16	19	4	2		18	13	16.3
17	28	4	1		7	13	17.3
18	20	8	1		7	9	16.6
19	28	8	4		15	10	18.6
20	28	8	3		6	12	14
21	20	4	1		14	13	19.2
22	26	8	3		not done	not done	17.2
23	18	3	3		3	not done	17.4
24	30	2	2		not done	not done	18
25	24	2	3		6	not done	17.2
26	32	4	3		6	not palpable	17.5
27	19	not done	3		10	not done	17.6
28	19	12	3		7	15	12.9
29	24	4	3		8	14	20
30	20	4	6		12	14	17.3
31	23	12	3		7	14	15.4
32	18	12		3	9	14	17.2
33	20	4	5		12	14	17.3
34	22	12	4		8	11	15.6
35	22	2		1	10	13	16.4
36	22	8	2		16	12	17.4
37	23	12		2	5	14	18.7
38	18	8	2		5	10	14.1
39	20	3	5		11	12	16.3
40	25	4	3		4	13	15.6
41	26	4		1	19	12	19.5
42	20	8	1		12	11	18.8
43	24	28	1		11	19	18.1
44	40	4	3		11	8	15
45	22	4	2		13	6	14.9
46	22	4	2		4	12	19.7
47	21	8	3		4	12	15.2
48	32	4	1		4	13	17.6
49	22	3	2		15	13	18.4
50	21	16	3		6	12	19.4
51	28	8	4		13	15	17.7
52	25	6	not done		17	12	18.8
53	28	4	2		12	14	19.7
54	18	8	5		10	15	15
55	20	20	3		9	14	14.2
56	22	16	3		13	16	17.7
57	22	6		3	13	15	15.6
58	25	4	3		9	6	17.3
59	23	3	6		15	17	17.9
60	18	4	3		14	not done	15

Duration of illness is defined as the number of weeks since the patients noticed symptoms associated with visceral leishmaniasis, such as fever, and/or enlarged abdomen (as a sign of enlarged spleen or liver).

**Table 2:** Intestinal parasite distribution amongst VL patients.

Patients	Hook worms	*S*. *mansoni*	*A. lumbricoides*	*T. Trichiura*	*E. histolytica*	*S. stercoralis*
1	-	-	-	-	+	-
2	-	-	-	-	-	-
3	-	-	-	-	-	-
4	-	-	-	-	-	-
5	+	-	-	-	-	-
6	+	-	-	-	-	-
7	+	+	-	-	-	-
8	-	-	-	-	-	-
9	-	-	+	-	-	-
10	-	-	-	-	-	-
11	+	-	-	-	-	-
12	+	-	-	-	-	-
13	+	-	+	-	-	-
14	-	-	-	-	-	-
15	-	-	-	-	-	-
16	-	-	-	-	-	-
17	-	-	-	-	-	-
18	-	-	-	-	-	-
19	+	-	-	-	-	-
20	-	-	-	-	-	-
21	+	-	-	-	-	-
22	-	-	-	-	-	-
23	-	-	+	-	-	-
24	-	-	-	-	-	-
25	-	-	-	-	-	-
26	-	-	-	-	+	-
27	-	+	-	-	-	-
28	-	-	-	-	-	-
29	-	-	-	-	-	-
30	-	-	-	-	-	-
31	-	+	-	-	-	-
32	-	-	-	-	-	-
33	-	-	-	-	-	-
34	-	-	-	-	-	-
35	-	+	+	-	-	-
36	+	-	-	-	-	-
37	-	-	-	-	-	-
38	-	-	+	-	-	-
39	-	-	-	-	-	-
40	+	-	-	-	-	-
41	+	-	-	-	-	-
42	+	-	-	-	-	-
43	-	-	-	-	-	-
44	-	-	-	-	-	+
45	+	-	-	-	-	-
46	+	-		-	-	-
47	-	-	+	+	-	-
48	-	-	-	-	-	-
49	-	-	-	-	-	-
50	-	-	+	-	-	-
51	+	-	+	-	-	-
52	-	-	-	-	-	-
53	-	-	-	-	-	-
54	-	-	-	-	-	-
55	-	-	-	-	-	-
56	+	-	-	-	-	-
57	-	-	-	-	-	-
58	+	-	-	-	-	-
59	-	-	-	-	-	-
60	+	-	-	-	-	-

We cannot exclude that we have underestimated strongyloidiasis due to the sensitivity of diagnostic tests based on parasitological examination, however, we did not have access to PCR or serological tests.

30.4% of the VL patients IP+ and 22.7% of VL patients IP- presented with diarrhoea. Importantly, co-infection with intestinal parasites did not affect the treatment of VL patients, as all 29 IP+ VL patients were successfully treated in the same time frame and had a positive initial clinical cure.

### Haematological data

**Fig 2:**
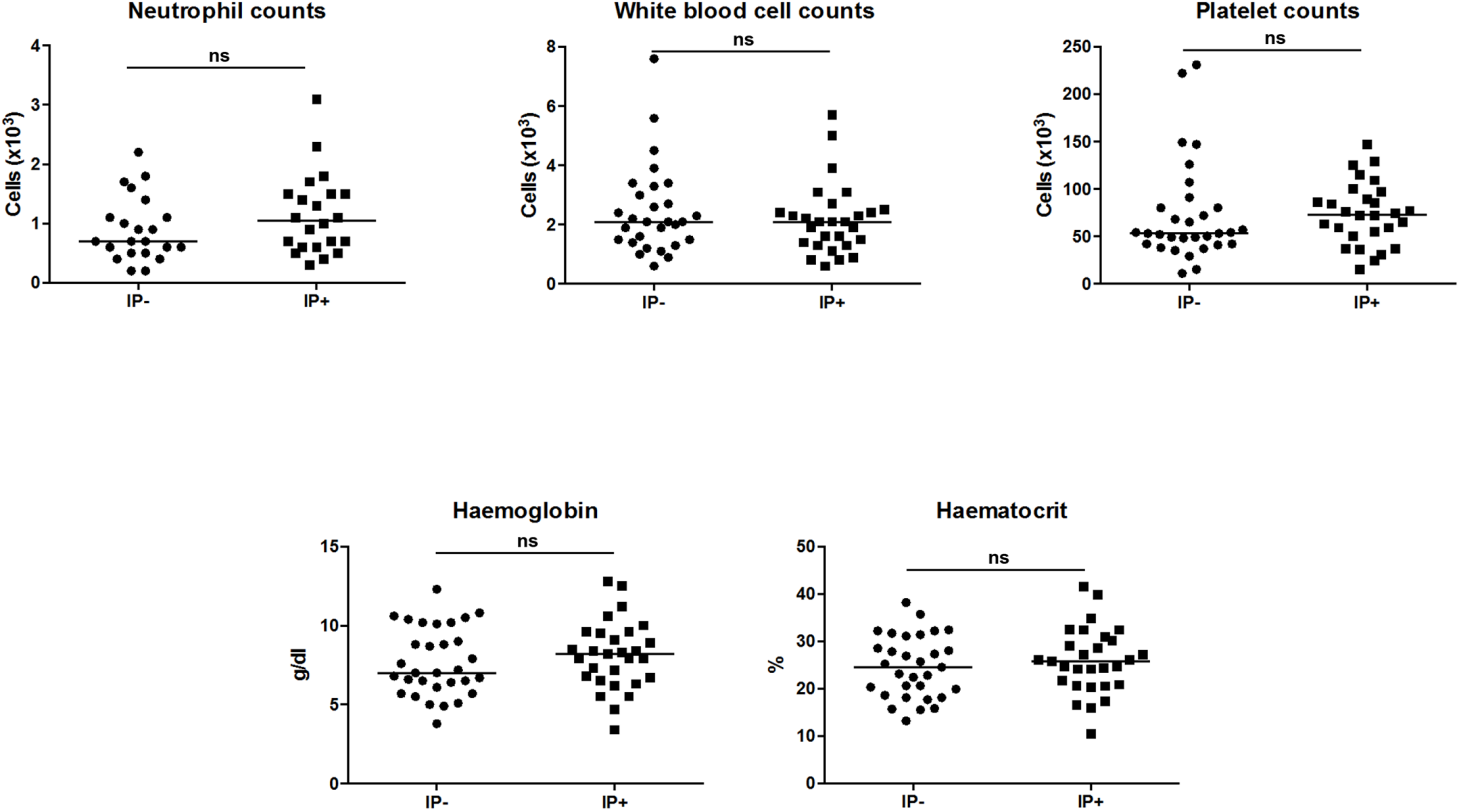
WBC = white blood cells; Plt = platelets; Hct = hematocrit; Hb = haemoglobin. Normal range: neutrophils (x10^3^)/μl = 2–7.5, platelets (x10^3^)/μl = 150–450; white blood cells (x10^3^)/μl = 4.5–10.5; Hct (%) = 35–60; hb (g/dl) = 11–18,Neutrophil (x10^3^) These parameters were measured in the blood of VL patients without intestinal parasite co-infections (IP-) and in VL patients co-infected with intestinal parasites (IP+), using a CELL-DYN 1800 Haematology Analyser, USA. Statistical significance was established using a Mann-Whitney test, ns = not significant (*p*<0.05).

Next, we assessed the haematological profile of the 2 cohorts of patients and as shown in Table 3 and Fig 2, VL patients presented with severe pancytopenia, and anaemia, as compared to healthy non-endemic controls (S1 Table). No significant differences (*p*>0.05) were observed in neutrophil, white blood cell (WBC) and platelet (Plt) counts, haemoglobin (Hgb) and haematocrit (Hct) between VL patients and VL patients co-infected with IP. The percentages of eosinophils in whole blood was low (<3) or undetectable in both groups, therefore no statistical differences could be evaluated.

**Table 3:** Comparison of complete blood count.

	IP-	IP+	*p* values
Neutrophils (x10^3^)/μl	0.7±0.1	1.1±0.1	0.2300
WBC (x10^3^)/μl	2.1±0.3	2.1±0.2	0.5705
Plt (x10^3^)/μl	53.0±9.5	73.0±6.2	0.2278
Hgb (g/dl)	7.0±0.4	8.2±0.4	0.4292
Hct (%)	24.5±1.2	25.8±1.3	0.4207

WBC = white blood cells

Plt = platelets

Hct = haematocrit

Hb = haemoglobin.

Normal range: neutrophils (x10^3^)/μl = 2–7.5; platelets (x10^3^)/μl = 150–450; white blood cells (x10^3^)/μl = 4.5–10.5; Hct (%) = 35–60; Hgb (g/dl) = 11–18.

These parameters were measured in the blood of VL patients without intestinal parasite co-infection (IP-) and in VL patients co-infected with intestinal parasites (IP+), using a CELL-DYN 1800 Haematology Analyser, USA.

Statistical significance was established using a Mann-Whitney test.

### Inflammatory response

**Fig 3:**
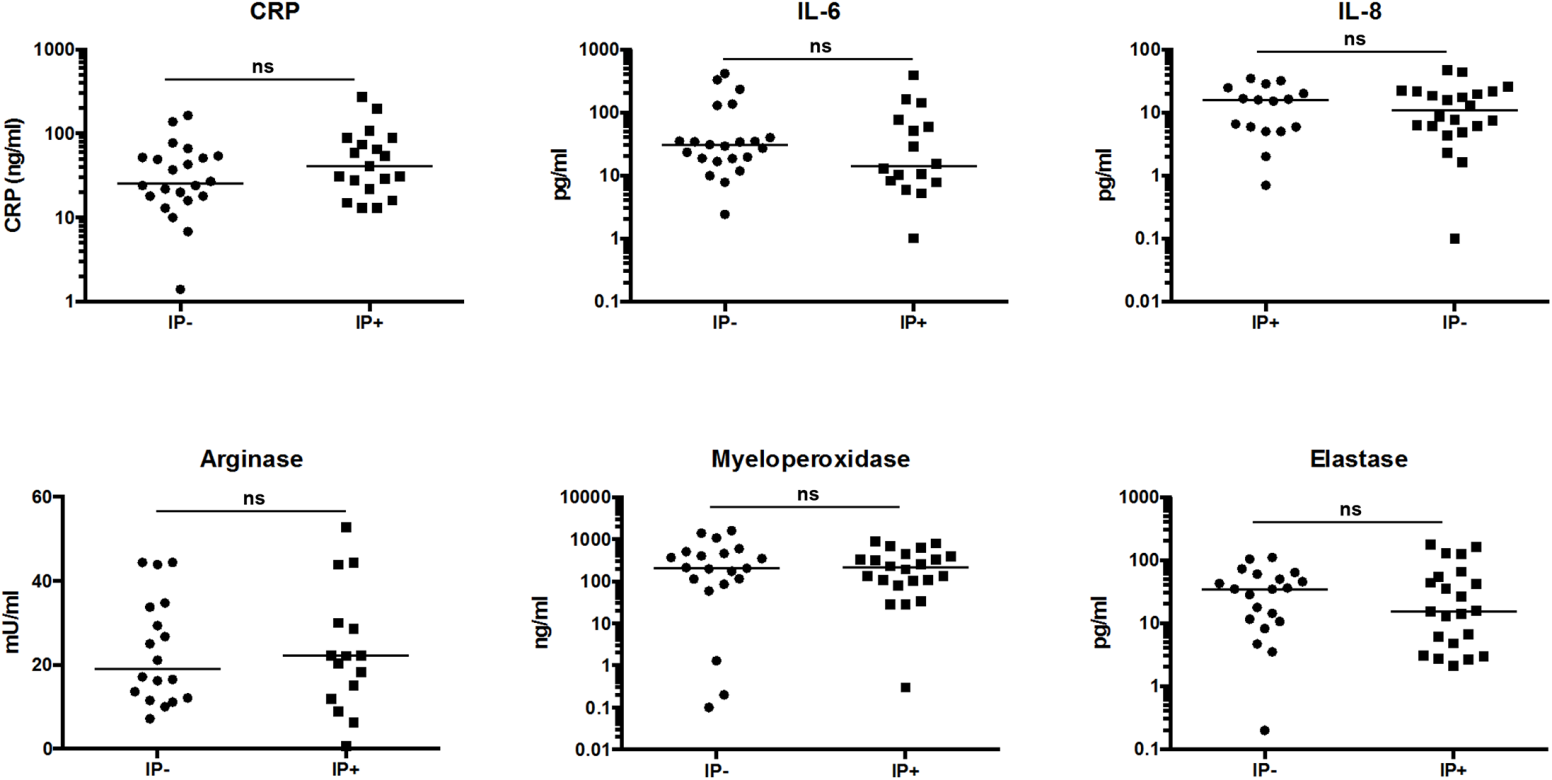
The plasma from VL patients without intestinal parasite co-infections (IP-) and in VL patients co-infected with intestinal parasites (IP+) was tested by ELISA (CRP, IL-6, IL-8, myeloperoxidase and elastase) or enzymatic activity (arginase) as described in Materials and Methods. Statistical significance was established using a Mann-Whitney test, ns = not significant (*p*<0.05).

VL is characterized by a strong systemic inflammation as measured by high levels of inflammatory cytokines in the plasma of these patients [8]. To assess whether co-infection of VL patients with IP had an impact on the systemic inflammation, the levels of CRP, IL-6 and IL-8 were measured in the plasma of the two groups of VL patients. The results confirm the strong inflammatory status, and as shown in Table 4 and Fig 3, no significant differences were observed. We also assessed the levels of arginase, myeloperoxidase and elastase in the plasma of the two groups of VL patients; these enzymes are all found in primary granules of neutrophils, which are the last granules to be released following activation of neutrophils. Results presented in Table 4 and Fig 3 show that the levels of these enzymes were similar in the plasma of the two groups of VL patients.

**Table 4:** Comparison of inflammatory markers.

	IP-	IP+	*p* values
CRP (μg/ml)	25.5±8.7	41.0±15.5	0.1736
IL-6 (pg/ml)	30.1±23.7	14.1±25.1	0.1715
IL-8 (pg/ml)	10.8±2.7	15.6±2.7	0.9942
Arginase (mU/ml)	19.1±3.0	22.1±3.8	0.9929
Myeloperoxidase (ng/ml)	208.5±102.7	212.5±55.1	0.6627
Elastase (pg/ml)	34.6±7.1	15.4±11.6	0.5578

The levels of CRP, IL-6, IL-8, myeloperoxidase and elastase were measured by ELISA and the levels of arginase activities by enzymatic assay, as described in Materials and Methods, in the plasma from VL patients without intestinal parasite co-infection (IP-) and in VL patients co-infected with intestinal parasites (IP+).

Statistical significance was established using a Mann-Whitney test.

### Th1, Th2 and regulatory cytokines

VL infections in human are characterised by high levels of IL-10 and IFN-γ [17]. To assess whether co-infection of VL patients with IP affects the systemic cytokine profile, we measured by Luminex and ELISA an array of different cytokines. Our results show that the Th1 (Table 5), Th2 (Table 6) and IL-10 (Table 7) cytokine profiles were similar in the plasma of both groups of VL patients.

**Table 5:** Comparison of Th1 cytokine levels.

	IP-	IP+	*p* values
IFN-γ (pg/ml)	433.0±107.1	774±125.7	0.1748
IL-2 (pg/ml)	0.0±4.3	0.0±1.6	0.5898
IL-12 (pg/ml)	0.8±0.9	0.1±15.7	0.5083

The levels of IFN-γ were measured by ELISA and the levels of IL-2 and IL-12 were measured by Luminex, as described in Materials and Methods, in the plasma from VL patients without intestinal parasite co-infection (IP-) and in VL patients co-infected with intestinal parasites (IP+).

Statistical significance was established using a Mann-Whitney test.

**Table 6:** Comparison of Th2 cytokine levels.

	IP-	IP+	*p* values
IL-4 (pg/ml)	Not detectable	Not detectable	Not applicable
IL-5 (pg/ml)	1.4±3.1	1.3±1.9	0.5086
IL-13 (pg/ml)	Not detectable	Not detectable	Not applicable

The levels of IL-4, IL-5 and IL-13 were measured by Luminex, as described in Materials and Methods, in the plasma from VL patients without intestinal parasite co-infection (IP-) and in VL patients co-infected with intestinal parasites (IP+).

Statistical significance was established using a Mann-Whitney test.

**Table 7:** Comparison of IL-10 levels.

	IP-	IP+	*p* values
IL-10 (pg/ml)	287.1±38.4	241.1±21.2	0.2274

The levels of IL-10 were measured by ELISA, as described in Materials and Methods, in the plasma from VL patients without intestinal parasite co-infection (IP-) and in VL patients co-infected with intestinal parasites (IP+).

Statistical significance was established using a Mann-Whitney test.

## Discussion

Our results show that the clinical and haematological data, inflammatory markers, cytokine profiles and BMI are similar in VL patients co-infected with intestinal parasites and VL patients with no intestinal parasites. The logistic regression model was used to test the association of IL-10, IL-8, IFN-γ, Plt, HCT, Hgb, WBC and BMI with IP. The IP variable was defined as positive response for at least one variable presented in Table 2. Due to low frequency of infections for the variables *T*. *trichiura*, *E*. *histolytica* and *S*. *stercoralis* (the last three variables reported in Table 2 with maximum number of infections of 1,2 and 2, respectively), co-infection patterns were investigated for hookworms, *S*. *mansoni* and *A*. *lumbricoides* using Fisher exact test (for the three possible pairs: hookworms and *S*. *mansoni*, hook worms and *A*. *lumbricoides* and *S*. *mansoni* and *A*. *lumbricoides*). In all cases it was found to be not significant.

All VL patients recruited at the LRTC presented with severe disease. Disease severity in VL patients has been associated with hepatosplenomegaly, low BMI, pancytopenia, anaemia and high parasite burden in splenic aspirates. Our results show hepatosplenomegaly in all but one patient (these measurements were not taken in two patients); that the majority of the VL patients were malnourished: 11 had a normal BMI (>18.5), 23 were moderately malnourished (BMI 16.5–18.4) and 26 were severely malnourished (<16.5) and there was no significant difference in BMI between VL patients with and without co-infection with intestinal parasites. Of note, we collected only one stool sample, however we maximised our chances to detect parasites by using three different techniques: direct saline wet mount, Kato-katz and "formol ether concentration" techniques. Furthermore, the neutrophil and white blood cell counts of both cohorts of VL patients were below the normal range. It is likely that the undetectable or very low percentages of eosinophils are due to the severe pancytopenia characteristic of VL patients. Finally, *Leishmania* parasites were present in the spleen or bone marrow aspirates of both patient cohorts. The results showing that VL patients are malnourished are in agreement with our previous results, showing that the large majority of VL patients are malnourished [18, 19]. Intestinal parasitic infections have long been associated with malnutrition in children [20, 21]: clinical manifestations of intestinal parasitic infection range from acute or persistent diarrhoea to dysentery, resulting in inflammation and nutrient malabsorption [22]. However, studies about intestinal parasitic infections and malnutrition in adults are less common and sometimes conflicting. For example, a study performed in Brazil showed that hookworm infection was associated with low BMI in adults [23]. But in a recent study performed in Ethiopia, the prevalence of undernutrition was similar in individuals infected with or without intestinal parasite infections [24]. In line with these results we show that the BMI of VL patients co-infected with IP were similar to those of VL patients. VL patients in the North West of Ethiopia are usually admitted with progressed, severe visceral leishmaniasis and since undernutrition and therefore a low BMI is characteristic of acute disease in patients with VL, [18, 19], any impact of intestinal parasite-related malnutrition might be hidden by the extent of malnutrition due to VL. Our results are in disagreement with those by Mengesha et al. [25], which show that VL patients were more likely to be severely malnourished when co-infected with IP. There are important differences between the recruitment of patients between our study and that of Mengesha et al., as we excluded other co-infection, such as TB, malaria and most importantly HIV; and indeed, we had previously shown that the BMI of HIV/VL patients was even lower as the BMI of VL patients [18]. In addition, we only considered patients with active intestinal parasitic infections, i.e. for example, patients with cysts of *Giardia* or *Entamoeba*, but no trophozoites, were excluded from our study. We did not find any significant differences in the median age, duration of illness, size of the spleen or liver and parasite grade in the spleen. Of note, in contrast to VL patients, none of the VL patients co-infected with IP had a maximum parasite grade of 5+ or 6+; however, the numbers of patients with high parasite burden are too low to make a significant conclusion. All the haematological data measured in the two groups were also similar and characteristic of VL patients [19].

VL is associated with high levels of systemic inflammation [8]. We have recently shown that neutrophils, which are major players in the induction and maintenance of inflammation, are highly activated and have degranulated, as shown by increased levels of arginase, myeloperoxidase and elastase in the plasma of VL patients [26]. These results suggested that the elevated levels of arginase, MPO and elastase are markers of a severe and systemic inflammatory response that is at least in part caused by high neutrophil activation. In our two cohorts of IP+ and IP- VL patients, the levels of arginase activity, myeloperoxidase and elastase were comparable. Furthermore, in addition to their potential role as markers of inflammation, these enzymes could also exacerbate immunopathological processes and therefore disease severity: arginase has been shown to play a role in T cell suppression [19] and in *Leishmania* parasite replication [14]; and both myeloperoxidase and elastase have the potential to affect parasite survival [27–29]. However, our results suggest that co-infection of VL patients with intestinal parasites does not impact on the levels of these three enzymes in the plasma.

Since parasitic infections are associated with an increased Th2-type response, we anticipated that the Th2 cytokines might be increased in VL patients co-infected with IP and that this might affect inflammation, but as shown by the similar levels of inflammatory mediators, the levels of inflammation are not altered in VL patients co-infected with IP; and indeed the levels of IL-5 were similar and those of IL-4 and IL-13 were below the detection limits of the ELISA we used.

In contrast to experimental models of leishmaniasis [30], the cytokine profile in VL patients is not associated with a Th1 or Th2 type cytokine profile. Indeed, high levels of both IL-10 and IFN-γ in the plasma are a hallmark of VL patients [17], IL-4 has been shown to be increased in VL patients [31], but only minimally [32] or even undetectable [33] in other studies. In line with the parameters we measured in the two groups of patients, the cytokine profiles of VL patients co-infected with IP are similar to that of VL patients.

The lack of apparent differences in the parameters we measured between the two groups of patients is likely to be due to the severity of VL, which might mask any differences that might be driven by the presence of intestinal parasites. Indeed, our previous study has shown that VL is severe in patients admitted to the *Leishmania* Research and Treatment Center in Gondar, as the duration of illness was considerably longer in the patients from the North West of Ethiopia as compared to those from Bihar, in India [34]; and that all patients presented with hepatosplenomegaly, low BMI, pancytopenia, anaemia and high parasite burden in splenic aspirates.

Further studies would benefit from a better characterization of the intestinal parasites in VL patients by using additional techniques, such as PCR, serological tests, as well as quantification of the load of each parasite and the collection of three consecutive samples.

In summary, our results show that co-infection of VL patients with IP does not affect VL disease severity, since clinical, haematological data and the treatment outcome are not altered by co-infection; in addition, none of the immunological markers we measured were different. However, in the light of the continuing debate about deworming [35], these results should be taken with care; our cohort of VL patients suffer from a highly debilitating disease, that is fatal if left untreated, and it might be therefore difficult to accurately dissect the contribution of a less severe disease, such as soil transmitted helminths. Notably, we did not address the medical conditions of these patients during follow-up, and whereas the relapse rate of VL patients is low [36] in this setting, we cannot exclude that these relapses might be linked to the presence of intestinal parasites. Interestingly, one recent study show that intestinal helminth infections, but not protozoan parasites, had a deleterious impact on the clinical course of cutaneous leishmaniasis (CL) caused by *Leishmania braziliensis*, as shown by increased frequency of mucosal lesions, poorer and longer response to therapy in co-infected CL patients [37]. Importantly, more work is needed to determine whether infection with an intestinal parasite prior to acquiring *Leishmania* parasites might lead to increased risks of developing leishmaniasis.

## Supporting information

S1 Checklist STROBE checklist. (DOCX)

S1 Table Complete blood count of healthy controls. WBC = white blood cells; Plt = platelets; Hct = hematocrit; Hb = haemoglobin. Normal range: neutrophils (x10^3^)/μl = 2–7.5; platelets (x10^3^)/μl = 150–450; white blood cells (x10^3^)/μl = 4.5–10.5; Hct (%) = 35–60; Hgb (g/dl) = 11–18. These parameters were measured in the blood of healthy non-endemic controls (n = 42) using a CELL-DYN 1800 Haematology Analyser, USA.
(DOCX)
