# Supplementary material for: Disease severity in patients with visceral leishmaniasis is not altered by co-infection with intestinal parasites
Source: PLoS Negl Trop Dis. 2017 Jul 21;11(7):e0005727. doi: 10.1371/journal.pntd.0005727 (PMC5540614; doi:10.1371/journal.pntd.0005727)
Supplement: S1 Table — WBC = white blood cells; Plt = platelets; Hct = hematocrit; Hb = haemoglobin. Normal range: neutrophils (x103)/μl = 2–7.5; platelets (x103)/μl = 150–450; white blood cells (x103)/μl = 4.5–10.5; Hct (%) = 35–60; Hgb (g/dl) = 11–18. These parameters were measured in the blood of healthy non-endemic controls (n = 42) using a CELL-DYN 1800 Haematology Analyser, USA. (DOCX) [file pntd.0005727.s002.docx]

**S1 Table. Complete blood count of healthy controls**

| Neutrophils (x10^3^)/μl | 3.5±0.3 |
| --- | --- |
| WBC (x10^3^)/μl | 6.7±0.3 |
| Plt (x10^3^)/μl | 271.5±9.6 |
| Hgb (g/dl) | 15.6±0.3 |
| Hct (%) | 48.5±0.5 |

WBC=white blood cells; Plt=platelets; Hct= hematocrit; Hb = haemoglobin.

Normal range: neutrophils (x10^3^)/μl = 2-7.5; platelets (x10^3^)/μl = 150-450; white blood cells (x10^3^)/μl = 4.5-10.5; Hct (%) = 35-60; Hgb (g/dl) = 11-18.

These parameters were measured in the blood of healthy non-endemic controls (n=42) using a CELL-DYN 1800 Haematology Analyser, USA.
